# Treatment Results in the Different Surgery of Intradural Extramedullary Tumor of 122 Cases

**DOI:** 10.1371/journal.pone.0111495

**Published:** 2014-11-05

**Authors:** Shaohui Zong, Gaofeng Zeng, Li Du, Ye Fang, Taihang Gao, Jingmin Zhao

**Affiliations:** 1 Department of Spine Osteopathia, the First Affiliated Hospital of Guangxi Medical University, Nanning, Guangxi, P.R. China; 2 College of Public Hygiene of Guangxi Medical University, Nanning, Guangxi, P.R. China; 3 Department of Orthopaedics Trauma and Hand Surgery, the First Affiliated Hospital of Guangxi Medical University, Nanning, Guangxi, P.R. China; Northwestern University, United States of America

## Abstract

**Study Design:**

A retrospective study of intradural extramedullary tumor.

**Objective:**

To compare the treatment results in the different surgeries of spinal intradural extramedullary tumor.

**Methods:**

The study retrospectively reviewed 122 patients. The minimally invasive surgery (MIS) group was divided into Group A (hemilaminectomy + tumor microscopic excision) and Group B (laminectomy + tumor microscopic excision + pedicle screw fixation). Meanwhile, the non-MIS group was divided into Group C (hemilaminectomy + tumor excision), Group D (laminectomy + tumor excision), and Group E (laminectomy + tumor excision + pedicle screw fixation). In order to study postoperative spinal stability, we simultaneously divided all of the subjects into three categories, namely Group HE: hemilaminectomy + tumor excision; Group LE: laminectomy + tumor excision; and Group LEPSF: laminectomy + tumor excision + pedicle screw fixation.

**Results:**

The MIS group exhibited fewer postoperative complications (p<0.05), better short-term clinical efficacy (p<0.05) and less non-surgical cost (p<0.05) than in non-MIS group. The rate of postoperative spinal instability in hemilaminectomy was lower than in laminectomy in a single spinal segment (p<0.05). The rate of postoperative spinal instability in laminectomy + pedicle screw fixation was lower than in hemilaminectomy and laminectomy in two or more spinal segments (p<0.05).

**Conclusion:**

In the case of appropriate surgical indications, minimally invasive surgery for intradural extramedullary tumor is a useful method that can successfully produce good clinical results and reduce non-surgical cost. In addition, pedicle screw fixation helps avoid spinal postoperative instability.

## Introduction

Intradural extramedullary tumors are very common in the osteopathic departments, accounting for approximately two-thirds of all primary intraspinal neoplasms [1]. As the tumors grow larger, the main outcome may be the compression of the spinal cord, which can cause back pain and worsening sensory and motor loss. The analyses of data from 25 cases revealed that the remaining spinal canal area was decreased to 20–30% of the normal spinal canal [2]. Therefore, the primary goal of the therapy for spinal intradural tumors is to decompress the spinal cord and remove it thoroughly without recurrence.

Nowadays, surgery is the effective approach for benign intradural extramedullary tumors [3]. For older people, minimally invasive surgery for benign intradural extramedullary tumor removal still represents the best therapeutic option [4], [5]. Although a conventional approach without a microscope can have a good curative effect, several drawbacks still exist, including greater intraoperative blood loss and a higher risk of injury to the spinal cord or nerve roots. With the development of technology, manipulation with a microscope could avoid these problems as far as possible. When removing spinal intradural extramedullary tumors by MIS or non-MIS, the damage to the anatomical structure of the posterior column of the spine will affects postoperative spinal stability.

This study reviewed a series of 53 consecutive patients with spinal intradural extramedullary tumors who underwent MIS and 69 consecutive patients with spinal intradural extramedullary tumors who underwent non-MIS. The purpose of this retrospective study is to compare the treatment results between MIS and non-MIS in these cases of spinal intradural extramedullary tumor and to analyze the postoperative spinal stability and health economics arising from the different surgical techniques.

## Materials and Methods

### Ethical statement

All study procedures were reviewed and approved by the Institutional Ethics Review Board at the First Affiliated Hospital of Guangxi Medical University and conducted according to the principles expressed in the Declaration of Helsinki. Informed consent was exempted by the board due to the retrospective nature of this research.

### Patient characteristics

122 patients who underwent surgical resection of intradural extramedullary tumors were retrospectively analyzed from January 2010 to June 2012. Data were obtained from the departments of surgery of six hospitals in the south of China. The ages of patients ranged from 15 to 82 years, with a mean age of 42 years. Symptom duration before surgery ranged from 2 months to 9 years, with a mean symptom duration of 31.2 months. The following inclusion criteria were used: 1) all of the subjects had intradural extramedullary tumor; 2) all of the subjects exhibited integrity of the spine and spinal stability before surgery; 3) clinical diagnosis was determined by physical examination, magnetic resonance imaging (MRI), and pathology; and 4) clinical and radiological follow-up was carried out for 12–60 months (average 28 months). Patients who underwent surgery again because of the recurrence of intraspinal tumor were excluded. The spinal levels of intradural extramedullary tumor were cervical vertebrae in 36 cases, cervicothoracic vertebrae in 2 cases, thoracic vertebrae in 39 cases, thoracolumbar vertebrae in 3 cases, and lumbosacral vertebrae in 42 cases.

### Surgical methods

All the patients were placed in the prone position under general anesthesia and a posterior median approach was chosen. The choice of surgical procedure depended on the location and size of the tumor. Hemilaminectomy was adopted when the tumor was small or located laterally or dorsolaterally to the spinal cord. Laminectomy was adopted when the tumor was large or located ventrally to the spinal cord. Pedicle screw fixation was required after multilevel laminectomy. Based on the spinal level of tumor, we inserted the pedicle screws in above 1-below 1. The subjects were divided into two groups according to the type of differential surgery, namely the MIS group (53 cases) and the non-MIS group (69 cases). The MIS group was subdivided into two groups, as follows:

- Group A: hemilaminectomy + tumor microscopic excision, 19 cases; and
- Group B: laminectomy + tumor microscopic excision + pedicle screw fixation, 34 cases.

Meanwhile, the non-MIS group was subdivided into the following three groups:

- Group C: hemilaminectomy + tumor excision, 24 cases;
- Group D: laminectomy + tumor excision, 18 cases;
- Group E: laminectomy + tumor excision + pedicle screw fixation, 27 cases.

In order to study postoperative spinal stability, 122 patients were simultaneously divided into three categories, as follows:

- Group HE: hemilaminectomy + tumor excision, 43 cases;
- Group LE: laminectomy + tumor excision, 18 cases; and
- Group LEPSF: laminectomy + tumor excision + pedicle screw fixation, 61 cases.

### Clinical outcome evaluation

The preoperative neurological status and neurological status at the time of discharge were evaluated in terms of back pain, dysesthesia, dyskinesia, and sphincter disturbance. Each category was divided into three grades, namely improved, unchanged, and deteriorative. In the postoperative evaluation of recent clinical efficacy, improvement and no change in neurological status were regarded as effectivity and deterioration of neurological status was regarded as nullity.

The evaluation of the long-term clinical efficacy was carried out using the American Spinal Injury Association classification (ASIA) [6]. In the evaluation of long-term clinical efficacy at the time of final follow-up (12–60 months, average 28 months), improvement was regarded as effectivity, while no change and deterioration were regarded as nullity.

All of the patients underwent examination of lateral roentgenograms during follow-up every 6 months. A spinal instability was diagnosed when either of the following conditions was observed [7]: 1. Abnormal mobility: abnormal segmental motion of a vertebral body of more than 15°; and 2. abnormal slide: anterior or posterior slide of a vertebral body of more than 3 mm.

### Health economics analysis

The total costs of hospitalization included surgical and non-surgical costs. Internal fixation device cost was included in surgical cost. Non-surgical cost included drug cost and hospital bed cost. Because the patients were from different socioeconomic backgrounds, an accurate estimation of work loss-related costs was not possible and this item was excluded from the cost analysis.

### Statistical analysis

All data are presented as the mean ± standard error of the mean (SEM). The statistical analysis was performed using SPSS 16.0 (SPSS, Inc., Chicago, IL, USA). Enumeration data were tested by the chi-squared test. Measurement data were tested by analysis of variance and t tests. A value of p<0.05 (two-tailed) was considered statistically significant.

## Results

There was no significant difference in age, gender or the course of disease between the MIS group and non-MIS group (p>0.05; Table 1). This was the same among the five groups (Group A, Group B, Group C, Group D, Group E; p>0.05; Table 1) and among the three groups (Group HE, Group LE, Group LESPF; p>0.05; Table 1).

**Table 1:** Summary of baseline characteristics of intradural extramedullary tumor patients.

Groups	N	Age (year)	Male/Female	Course of disease(mouth)
MIS group	53	41.15±14.73	29/24	32.45±6.81
Non-MIS group	69	43.25±16.42	37/32	30.67±5.28
Group A	19	42.84±11.25	11/8	28.25±8.14
Group B	34	41.42±13.47	16/18	29.53±8.75
Group C	24	40.26±15.08	14/10	33.67±7.99
Group D	18	43.71±12.23	10/8	30.73±7.43
Group E	27	43.54±14.67	15/12	31.84±6.58
Group HE	43	40.87±12.54	21/22	29.72±4.73
Group LE	18	41.62±13.75	10/8	34.91±7.54
Group LESPF	61	43.92±15.68	35/26	33.14±9.36

There was no significant difference between the MIS group and Non-MIS group (p>0.05). This was the same among the five groups (Group A, Group B, Group C, Group D, Group E; p>0.05) and among the three groups (Group HE, Group LE, Group LESPF; p>0.05). Data are expressed as the mean ± standard deviation.

### Clinical results

In the MIS group (53 cases), postoperative pathologies showed schwannoma in 26 cases, meningioma in 12, dermoid cyst in 4, hemangioma in 3, and lipoma in 2. Among these, 41 tumors were totally removed, 10 were subtotally removed, and 2 were partially removed. Two complications were observed in the MIS group, including one case of infection and one case of myasthenia. Meanwhile, in the non-MIS group (69 cases), postoperative pathologies showed schwannoma in 29 cases, meningioma in 15, dermoid cyst in 12, arachnoid cyst in 9, enterogenous cyst in 3, and hemangioma in 1. Among these, 50 tumors were totally removed, 15 were subtotally removed, and 4 were partially removed. Twelve complications were observed in the non-MIS group, including six cases of leakage of cerebrospinal fluid, three cases of infection, two cases of myasthenia, and one case of localized pain.

There was no significant difference in the rate of tumor resection between the MIS group and non-MIS group (p>0.05). The estimated blood loss and hospital stay durations observed in the MIS group were significantly less than in the non-MIS group (p<0.05). The operating time observed in the MIS group was significantly longer than in the non-MIS (p<0.05). The rate of postoperative complication in the MIS group was lower than in the non-MIS group (p<0.05) (Table 2).

**Table 2:** Summary of the results for operating time, intraoperative blood loss, resection rate, duration of hospital stay, and complications.

Groups	N	Operating time(min)	Intraoperativeblood loss (ml)	Resectionrate (%)	Duration ofhospital stay(d)	Complications
MIS group	53	270.00±147.99★	307.14±185.13★	77.36▴	21.88±10.15★	2★
Non-MISgroup	69	173.26±81.36	540.08±282.15	72.46	26.42±11.60	12

This table shows the results for operating time, intraoperative blood loss, resection rate, duration of hospital stay, and complications in the MIS and Non-MIS group. “Resection rate” means the percentage of the cases of total resection of tumor. Data are expressed as the mean ± standard deviation.

★ *p*<0.05 vs. MIS group;

▴ *p*>0.05 vs. Non-MIS group.

As the neurological status was reevaluated at the time of discharge, the results were as follows: In the MIS group, postoperative neurological status improved or remained unchanged in 52 cases and worsened in 1 case (dysesthesia). In the non-MIS group, postoperative neurological status improved or remained unchanged in 59 cases and worsened in 10 cases (4 cases of back pain, 3 cases of dysesthesia, 2 cases of dyskinesia, 1 case of sphincter disturbance). Based on the results above, the short-term clinical efficacy in the MIS group was superior to that in the non-MIS group (p<0.05; Table 3).

**Table 3:** Analysis of short-term and long-term clinical efficacy.

Groups	N	Short-term clinical efficacy			Long-term clinical efficacy		
		Effectivity	Nullity	Rate ofeffectivity (%)	Effectivity	Nullity	Rate ofeffectivity (%)
MIS group	53	52	1	98.11★	51	2	96.22▴
Non-MIS group	69	59	10	85.50	64	5	92.75

This table shows the results for short-term and long-term clinical efficacy in the MIS and Non-MIS groups.

★ *p*<0.05 vs. Non-MIS group;

▴ *p*>0.05 vs. Non-MIS group.

According to ASIA, the results of the long-term clinical efficacy were observed as follows: In the MIS group, as the neurological status was reevaluated at the time of the final follow-up (12–60 months, average 28 months), neurological status improved in 51 cases and remained unchanged in 2 cases. In the non-MIS group, as the neurological status was reevaluated at the time of the final follow-up (12–60 months, average 28 months), neurological status improved in 65 cases, remained unchanged in 4 cases, and worsened in 1 case. Based on the results, there was no statistically significant difference between the MIS group and non-MIS group in terms of long-term clinical efficacy (p>0.05; Table 3).

### Health economics analysis

**Figure 1:**
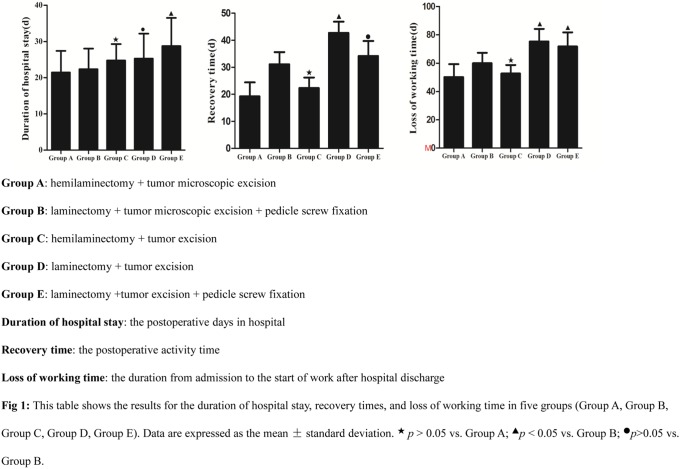
This table shows the results for the duration of hospital stay, recovery times, and loss of working time in five groups (Group A, Group B, Group C, Group D, Group E). Data are expressed as the mean ± standard deviation. ★p>0.05 vs. Group A; ▴p<0.05 vs. Group B; •p>0.05 vs. Group B. Group A: hemilaminectomy + tumor microscopic excision. Group B: laminectomy + tumor microscopic excision + pedicle screw fixation. Group C: hemilaminectomy + tumor excision. Group D: laminectomy + tumor excision. Group E: laminectomy + tumor excision + pedicle screw fixation. Duration of hospital stay: the postoperative days in hospital. Recovery time: the postoperative activity time. Loss of working time: the duration from admission to the start of work after hospital discharge.

There were no significant differences in the hospital stay durations, recovery times, or loss of working time between Group A and Group C (p>0.05). The hospital stay durations and loss of working time observed in Group B were significantly less than in Group E (p<0.05). There was no significant difference in recovery times between Group B and Group E (p>0.05). The recovery times and loss of working time in Group B were significantly less than in Group D (p<0.05). There was no significant difference in hospital stay durations between Group B and Group D (p>0.05; Table 4; Figure 1).

**Table 4:** Summary of results of duration of hospital stay, recovery time, loss of working time.

Groups	N	Duration of hospitalstay (d)	Recovery time (d)	Loss of working time (d)
A	19	21.42±10.36	19.29±8.92	50.13±15.76
B	34	22.35±9.83	31.12±7.79	59.98±12.83
C	24	24.76±7.81★	22.31±6.78★	52.69±10.33★
D	18	25.29±11.92•	42.72±7.25▴	75.38±15.22▴
E	27	28.73±13.47▴	34.18±9.6•	71.81±17.19▴

This table shows the results for the duration of hospital stay, recovery times, and loss of working time in five groups (Group A, Group B, Group C, Group D, Group E). “Recovery time” means the postoperative activity time. “Duration of hospital stay” means the postoperative days in hospital. “Loss of working time” represents the duration from admission to the start of work after hospital discharge**.** Data are expressed as the mean ± standard deviation.

★ *p*>0.05 vs. Group A;

▴ *p*<0.05 vs. Group B;

• *p*>0.05 vs. Group B.

In terms of the costs of surgery, there is no significant difference between Group A and Group C (p>0.05), which was same between Group B and Group E (p>0.05). Group B was significantly higher than Group D (p<0.05). Meanwhile, in relation to non-surgical costs, those in Group A were significantly less than those in Group C (p<0.05). In addition, those of Group B were significantly less than those of Group E (p<0.05), while those of Group B were significantly less than those of Group D (p<0.05). Regarding the total costs of hospitalization, the total costs of hospitalization between Group A and Group C showed no significant difference (p>0.05), which was same between Group B and Group E (p>0.05). On the other hand, those of Group D were significantly less than those of Group B (p<0.05) (Table 5).

**Table 5:** Summary of various expenses.

Groups	N	Surgical cost (CNY)	Non-surgical cost (CNY)	Cost of hospitalization (CNY)
A	19	13467±7682	7782±3129	20971±12318
B	34	29314±12854	6459±2743	34354±13214
C	24	10525±9078★	10974±5607▴	21509±8842★
D	18	11628±7216•	11691±8329•	22319±11327•
E	27	27612±10722▪	9621±6031•	37234±11999▪

This table shows the analysis of health economics for the five surgical approaches. Data are expressed as the mean ± standard deviation.

★ *p*>0.05 vs. Group A;

▴ *p*<0.05 vs. Group A;

• *p*<0.05 vs. Group B;

▪ *p*>0.05 vs. Group B.

### Study of postoperative spinal stability

The rate of postoperative spinal instability in Group HE and Group LEPSF were significantly lower than that in Group LE in a single spinal segment (p<0.05). The rate of postoperative spinal instability between Group HE and Group LEPSF showed no significant difference in a single operative segment. The rate of postoperative spinal instability in Group LEPSF was significantly lower than those in Group HE and Group LE in two or more spinal operative segments (p<0.05). The rate of postoperative spinal instability between Group HE and Group LE showed no significant difference in two or more operative segments (p>0.05; Table 6).

**Table 6:** Summary of postoperative spinal stability in different segments.

Groups	1 segment			2 segments			>2 segments		
	stable	instable	Rate of stability (%)	stable	instable	Rate of stability (%)	stable	instable	Rate of stability (%)
HE	21	0	100.00	8	5	61.54	3	6	33.33
LE	4	2	66.67★	3	3	50.00•	1	5	16.67•
LEPSF	22	1	95.65^▴•^	26	2	92.86^★▴^	9	1	90.00^★▴^

This table shows the results of postoperative spinal stability of various surgical approaches in different segments. Abbreviations: HE: hemilaminectomy + tumor excision; LE: laminectomy + tumor excision; LEPSF: laminectomy + tumor excision + pedicle screw fixation.

★ *p*<0.05 vs. Group HE;

▴ *p*<0.05 vs. Group LE;

• *p*>0.05 vs. Group HE.

## Discussion

Intradural extramedullary tumors account for two-thirds of primary spinal tumors [8]. Most intradural extramedullary tumors are benign, and they exhibit no specific symptoms. Radicular pain and worsening sensory and motor loss are common manifestations. Therefore, most of the patients are wrongly diagnosed with cervical spondylopathy or intervertebral disk herniation. MRI is very crucial to confirm the diagnosis of intradural extramedullary tumors. Once the diagnosis is confirmed, the best treatment for nonmalignant intradural extramedullary tumor is surgery. The goal of surgery is complete surgical resection while preserving spinal stability, without worsening the preoperative neurological status.

All the processes of conventional surgical tumor resection are carried out without using microscope, which may lead to a greater likelihood of incomplete tumor resection, as well as more damage to the spinal cord and vessels surrounding the spinal canal. With the improvement of medical devices, surgeons are increasingly using microscopy to perform surgical tumor resection. MIS can provide a clearer visual operative field and more delicate operative maneuvers, which can avoid the damage to the spinal cord and peripheral nerves as far as possible, reduce intraoperative blood loss and postoperative complications, and increase the rate of complete removal of tumors. It was reported that MIS allowed removal of the tumor with minimal impairment from cutting of nerve fibers at the nerve root [9]. Regarding intramedullary tumors, MIS made it possible to reduce postoperative mortality from 38.5 to 4.5%, exclude various complications (postoperative liquorrhea, meningitis), restore the disturbed functions of the spinal cord fully, and prolong postoperative remission and the patient’s survival [10]. After total resection, restoration of full spinal cord function can happen in limited numbers of cases.

In our present study, the MIS group had the advantages of less estimated blood loss, shorter hospital stays, and fewer postoperative complications than those were observed in the non-MIS group (p<0.05). The short-term clinical efficacy of the MIS group was superior to that of the non-MIS group (p<0.05). However, there was no significant difference in the resection rates of tumors or long-term clinical efficacy between the MIS group and non-MIS group (p>0.05). Micromanipulation achieved less intraoperative blood loss than in the non-MIS group through avoiding accidental injury of epidural and spinal cord vessels as much as possible and precisely intercepting arteries supplying the tumor. In the MIS group, micromanipulation prevented as many complications as possible, such as postoperative infection and cerebrospinal leakage; this is because micromanipulation could reduce intraoperative blood loss and trauma to tissues around surgical site. What’s more, a small mount of intraoperative blood loss has modesty impact on patient’s body immunity and the duration before functional exercises on limb function, which is helpful to reduce postoperative infection effectively. Although non-MIS manipulation did not directly damage the anatomical structure of the spinal cord, it increased the damage to the vessels supplying the spinal cord than in the MIS group, which could lead to less-than-ideal postoperative spinal function recovery and short-term clinical efficacy.

Although we cannot use health economics analysis to determine the best treatment for any particular disease, we can get some useful references for surgical options. In this study, the non-surgical cost in MIS is less than in non-MIS (p<0.05), this is because MIS can result in the less intraoperative blood loss, lower postoperative complications, shorter durations of hospital stay and better short-term clinical efficiency. As surgical cost takes a great proportion in the total cost of hospitalization, the total costs of hospitalization between Group A (hemilaminectomy + tumor microscopic excision) and Group C (hemilaminectomy + tumor excision) showed no significant difference, which was same between Group B (laminectomy + tumor microscopic excision + pedicle screw fixation) and Group E (laminectomy + tumor excision + pedicle screw fixation). Because the costs of the internal fixation device must be taken into consideration, the total cost of hospitalization in Group B (laminectomy + tumor microscopic excision + pedicle screw fixation) is not less than in Group D (laminectomy + tumor excision). The health economics analysis was based on data from six hospitals, and therefore, may not be representative of costs at other hospitals.

In our study, we found that hemilaminectomy for intradural extramedullary tumor had better postoperative spinal stability than laminectomy with a single spinal segment. However, conventional laminectomy is still widely used for the surgical removal of spinal intradural extramedullary tumor, which can offer more familiar exposure and a wider view of the surgical field to surgeons than in hemilaminectomy. In contrast, some serious drawbacks of laminectomy still exist, such as spinal instability, epidural fibrosis, the absence of osseous protection for the spinal cord, and postoperative dorsal pain [11], [12].

The removal of the posterior lamina, spinous process, paravertebral muscles, supraspinous ligament, interspinous ligament, and ligamentum flavum is needed in the surgical corridor of laminectomy, which causes great damage to the posterior column of the spine, in turn jeopardizing spinal stability [13], [14], [15], [16]. In an effort to maintain spinal stability, hemilaminectomy avoids damage to the posterior structure of the vertebral column by preserving the supraspinous ligament, interspinous ligament, and paravertebral muscle of the opposite side. Therefore, hemilaminectomy has less impact on spinal biomechanics [17], [18]. On the other hand, the disadvantage of hemilaminectomy is the narrow surgical corridor, which increases the risk of injury to spinal cord [19]. In our study, there was no statistical difference about spinal stability between hemilaminectomy and laminectomy in two or more operative segments of spine. Another result was that laminectomy + pedicle screw fixation showed better postoperative spinal stability than hemilaminectomy and laminectomy in two or more spinal operative segments. It is generally known that pedicle screw fixation offers a significant biomechanical advantage [20], and is superior to other techniques regarding the promotion of mechanical strength [21], [22]. As a result, great internal stability is observed.

In this study, we have found that MIS is advantageous in the removal of intradural extramedullary tumor, this is because MIS can reduce intraoperative blood loss, duration of hospital stay, postoperative complications, non-surgical cost and have better short-term clinical efficacy compared with non-MIS. In addition, in two or more spinal operative segments, pedicle screw fixation helps avoid spinal postoperative instability. However, we have not investigated if the MIS is helpful to postoperative stability in this study. Besides that, the follow-up time and preoperative radiographic assessment of spinal stability need to be further improved in our future study. In summary, the study provides some insight to help surgeons choose the appropriate surgical method for intradural extramedullary tumor.
